# The Association of *Macavirus* and Ovine Gammaherpesvirus 2 with Pneumonia in Beef Cattle from Mato Grosso, Brazil

**DOI:** 10.3390/pathogens14090945

**Published:** 2025-09-18

**Authors:** Julia Raisa Ximenes Figueiredo, Flavia Helena Pereira Silva, Juliana Torres Tomazi Fritzen, Beatriz Martins Machado, Fernanda Pinto Ferreira, Karina Rodrigues Gomes Ferreira, Sébastien Buczinski, Amauri Alcindo Alfieri, Selwyn Arlington Headley

**Affiliations:** 1 Programa de Pós-Graduação em Biociência Animal, Universidade de Cuiabá, Cuiabá 78065-900, Brazil; raisa_norris@hotmail.com (J.R.X.F.); karinargomes26@gmail.com (K.R.G.F.); 2 Laboratory of Animal Pathology, Department of Preventive Veterinary Medicine, Universidade Estadual de Londrina, Londrina 86057-970, Brazil; flaviahelena.pereira@uel.br; 3 Laboratory of Animal Virology, Department of Preventive Veterinary Medicine, Universidade Estadual de Londrina, Londrina 86057-970, Brazil; jufritzen@uel.br (J.T.T.F.); beatriz.mmachado@uel.br (B.M.M.); alfieri@uel.br (A.A.A.); 4 Zoonoses and Epidemiology, Department of Preventive Veterinary Medicine, Universidade Estadual de Londrina, Londrina 86057-970, Brazil; fpferreira@uel.br; 5 Département des Sciences Cliniques, Faculté de Médecine Vétérinaire, Université de Montréal, St-Hyacinthe, QC J2S 2M2, Canada; s.buczinski@umontreal.ca; 6 Multi-User Animal Health Laboratory (LAMSA), Department of Preventive Veterinary Medicine, Universidade Estadual de Londrina, Londrina 86057-970, Brazil

**Keywords:** bovine respiratory disease, concomitant infections, interstitial pneumonia, malignant catarrhal fever virus, pulmonary disease, subclinical infections

## Abstract

This study investigated the possible occurrence of pulmonary disease in beef cattle from 13 municipalities within the State of Mato Grosso (MT), Brazil. The state of MT is a leading player in beef cattle production in Brazil, but with comparatively few data relative to the occurrence of pulmonary disease or ovine gammaherpesvirus 2 (OvGHV2)-related infections in cattle. Pulmonary samples from 44 beef cattle, with ages ranging between 18 and 28 months, were collected during slaughter and processed to determine the patterns of pulmonary lesions. Additionally, duplicate fragments were used in immunohistochemical (IHC) assays designed to detect malignant catarrhal fever (MCFV) antigens and in molecular assays to amplify 10 agents associated with the development of bovine respiratory disease (BRD). Interstitial pneumonia (IP) was diagnosed in most of the lungs (98%; 43/44) evaluated from all municipalities. MCFV antigens were detected in 37% (16/43) of the animals with IP. Only four pathogens were amplified by molecular assays within the lungs of cattle with IP: OvGHV2 (23%; 10/43), bovine viral diarrhea virus (12%; 5/43), bovine coronavirus (7%; 3/43), and *Mannheimia haemolytica* (2%; 1/43). The nucleic acids of bovine respiratory syncytial virus, bovine alphaherpesvirus 1, bovine parainfluenza virus 3, *Pasteurella multocida*, *Histophilus somni*, and *Mycoplasmopsis* (*Mycoplasma*) *bovis* were not amplified. Singular infections in cattle from municipalities were associated with MCFV (*n* = 3) and OvGHV2 (*n* = 2), while OvGHV2 occurred in all dual and triple infections. However, in four animals with IP, none of the disease pathogens identified were detected. Statistically, MCFV antigens were more frequently (*p* = 0.048) detected in the lungs of female (75%; 12/16) cattle with IP relative to males (25%; 4/16). Additionally, there was a positive correlation (*p* < 0.001) between the IHC detection of MCFV antigens within the bronchial epithelium and the epithelium of peribronchial glands of cattle with IP. This is the first study to statistically demonstrate that female cattle are at greater risk of developing MCFV-related infections as compared to male animals. The detection of OvGHV2 in singular and multiple infections during this investigation supports earlier studies that associate this pathogen with the development of pulmonary disease in cattle, indicating that OvGHV2 can contribute to the etiology of IP. Additionally, the detection of OvGHV2-induced infections in asymptomatic cattle suggests that all animals were subclinically infected, confirming that subclinically OvGHV2-induced infections may be widespread in ruminants from Brazil. Furthermore, the occurrence of atypical interstitial pneumonia cannot be discarded, particularly in animals with IP but without any associated pathogen. These initial findings suggest the need for a more elaborate investigation to understand the dynamics of pulmonary disease within this state.

## 1. Introduction

*Macavirus* is a group of unique organisms within the subfamily *Gammaherpesvirinae*, family *Orthoherpesviridae* [1], that can broadly be divided based on their known association with the development of malignant catarrhal fever (MCF) in susceptible mammalian populations. *Macavirus* associated with MCF have the 15A antigenic epitope [2], are highly conserved at the genomic and sequence levels [3], and are collectively referred to as the MCF virus (MCFV) complex [3,4]. Of the MCFV that produces MCF in susceptible hosts, ovine gammaherpesvirus 2 (*Macavirus ovinegamma* 2; OvGHV2) and alcelaphine gammaherpesvirus 1 (*Macavirus alcelaphinegamma* 1; AlGHV1) [1] are the most widely studied due to their economic and epidemiological importance [5,6,7,8]. OvGHV2 produces sheep-associated MCF worldwide, while AlGHV1 induces wildebeest-associated MCF principally in ruminants from Africa [5,7,9]. Although there is evidence of the circulation of an unknown *Macavirus* that is associated with infections in ruminants from Brazil [10,11], thus far, OvGHV2 is the only MCFV known to produce MCF in ruminants from this continental nation [7].

Pneumonia in cattle is part of the bovine respiratory disease (BRD) complex that is caused by viral and bacterial agents, including bovine viral diarrhea virus (BVDV), bovine respiratory syncytial virus (BRSV), bovine alphaherpesvirus 1 (BoAHV1), bovine coronavirus (BCoV), bovine parainfluenza virus 3 (BPIV3), *Mannheimia haemolytica*, *Pasteurella multocida*, *Mycoplasmopsis* (*Mycoplasma*) *bovis*, and *Histophilus somni* [12,13,14,15]. Additionally, there is adequate evidence to demonstrate that OvGHV2 may be an inductor of pneumonia in cattle [16,17,18]. In addition to viral and bacterial pathogens, BRD is also associated with abrupt alterations in management practices, weather, and several stress-induced conditions, as well as host-related problems [12,19]. Moreover, atypical interstitial pneumonia (AIP) is frequently associated with respiratory diseases in feedlot cattle [20]. Although the exact economic effects of BRD on the cattle industry in Brazil are currently unknown, it was estimated that the effects of mortality and morbidity due to BRD may impact the local economy annually by 6.3 million and 5.5 million USD, respectively [21].

The state of Mato Grosso (MT) is located within Midwestern Brazil, and forms part of the Cerrado biome, which contributes to 23% of the Brazilian territory [22] and has the largest population of cattle [23]. In 2023, the Midwestern region of Brazil had 32% (76,698,859/238,626,442) of all cattle from Brazil, with MT being the largest producer of cattle nationwide, where 14% (33,994,004/238,626,442) of cattle were reared in that year [24]. The average slaughter weight of cattle in the Cerrado biome varies between 380 and 480 kg [23]. However, the published data relative to the occurrence of pulmonary diseases of cattle within the state of MT is lacking. In contrast, most descriptions of BRD in Brazil originated from the States of Minas Gerais, Paraná, and São Paulo [12]. Additionally, there are relatively few studies that have identified infections due to OvGHV2 in MT [25,26,27]. A retrospective study based on the reports from a veterinary diagnostic laboratory in MT revealed that SA-MCF was diagnosed in only 0.7% (8/1124) of all cattle evaluated during a 9-year period [28]. In addition, a serological investigation described elevated levels of seropositivity for BVDV, BoAHV1, BRSV, and BPIV3 in steers on arrival at beef cattle feedlots in MT [29]. Furthermore, additional data relative to the occurrence of pulmonary disease in cattle from MT were not identified when major English and Latin databases were examined. Collectively, these findings suggest that the documented information as to the occurrence of pulmonary disease in cattle from this state is scarce. Consequently, understanding the dynamics of pulmonary and OvGHV2-related diseases in cattle from the state of MT is of fundamental importance, considering the contribution of this state towards the cattle rearing industry in Brazil.

Therefore, the objectives of this study were: (1) associate the patterns of pneumonia with specific infectious disease pathogens of BRD; (2) identify possible risk factors associated with pulmonary infections by *Macavirus* within the state of MT; (3) provide additional information as to the occurrence of pulmonary infections by OvGHV2 and/or *Macavirus* in cattle; and (4) to obtain preliminary information on the occurrence of pulmonary lesions in beef cattle from MT.

## 2. Materials and Methods

### 2.1. Study Location, Animals, and Sample Collection

Pulmonary samples (*n* = 44) were randomly collected from the lungs of beef cattle at 10 slaughterhouses under either Federal, State, or Municipal Inspection Services from the Southeastern and Northeastern mesoregions of the state of MT, between June 2023 and January 2024. All cattle originated from 13 municipalities that were within the proximity of these slaughterhouses. Cattle from these regions were predominantly of the Nelore breed, of both sexes, with ages varying between 18 and 28 months, all animals were immunized against foot-and-mouth-disease and clostridiosis, while all females were immunized against brucellosis. Furthermore, cattle from these farms were reared either by the extensive, semi-extensive, or intensive production systems [30]. All cattle had excellent body scores and were slaughtered at an average of 350 kg live weight.

All pulmonary samples were collected in duplicate: one part was immersed in 10% buffered formalin solution and routinely processed for histological evaluation with the Hematoxylin and eosin stain. The remaining half of each tissue sample was maintained at −80 °C until used in molecular assays. This dual evaluation had two objectives: ensure that both morphological and molecular aspects of pulmonary disease could be thoroughly evaluated and to enable correlation between the histopathological findings and the presence of specific pathogens. Collectively, this approach provides a better understanding of the infectious dynamics in the cattle populations evaluated.

### 2.2. Histopathological and Immunohistochemical Analyses

Histopathology was done to identify the pattern of pulmonary alteration observed in each sample. The pattern of pulmonary lesions was categorized as either bronchopneumonia (fibrinous or suppurative), interstitial, granulomatous, or embolic pneumonia [31].

Selected formalin-fixed paraffin-embedded (FFPE) tissue sections were used in immunohistochemical (IHC) assays designed to detect MCFV antigens with the 15A IHC assay [32]. Additionally, the presence of these antigens was associated with the pattern of pulmonary alteration identified by histopathology. Positive controls consisted of the utilization of FFPE sections known to contain OvGHV2 antigens derived from previous studies [32,33]. Negative controls consisted of FFPE tissue sections that did not contain neither MCFV or OvGHV2 antigens and by replacing the primary antibody with its diluent. Positive and negative controls were included in each IHC assay.

### 2.3. Molecular Investigation of Pulmonary Disease Pathogens of Cattle

The nucleic acids from all pulmonary fragments maintained at −80 °C were extracted by using a combination of the phenol/chloroform/isoamyl alcohol and silica isothiocyanate/guanidine methods [34,35] and then used in molecular assays designed to amplify the DNA/RNA of bacterial and viral pathogens associated with the development of BRD. These pathogens included BVDV, BRSV, BoAHV1, BCoV, BPIV3, OvGHV2, *M. haemolytica*, *P. multocida*, *H. somni*, and *M. bovis*. A list of the target genes and the amplicon sizes of the agents used during this investigation is provided (Supplementary Table S1). Positive controls consisted of the utilization of nucleic acids of these agents derived from previous investigations [18,33]. The negative control consisted of ultrapure water. Positive and negative controls were included in each molecular assay.

All PCR products were separated by electrophoresis in 2% agarose gels, stained with ethidium bromide, and examined under ultraviolet light. The products derived from all molecular assays were purified using the PureLink^®^ Quick Gel Extraction and PCR Purification Combo Kit (Invitrogen^®^ Life Technologies, Carlsbad, CA, USA), quantified by using a Qubit^®^ Fluorometer (Invitrogen^®^ Life Technologies, Eugene, OR, USA), and submitted to direct sequencing in both directions with the forward and reverse primers used in the respective molecular assays in an ABI3500 Genetic Analyzer sequencer with the BigDye Terminator v3.1 Cycle Sequencing Kit (Applied Biosystems^®^, Foster City, CA, USA).

Sequence quality analyses and consensus sequences were obtained using PHRED and CAP3 homepage (http://asparagin.cenargen.embrapa.br/phph, accessed on 31 March 2025), respectively. Similarity searches of the OvGHV2 tegument protein gene were performed with nucleotide (nt) sequences deposited in GenBank using the BLAST homepage (https://blast.ncbi.nlm.nih.gov/Blast.cgi accessed on 31 March 2025).

### 2.4. Types of Infections Detected in Municipalities of Mato Gross, Brazil

The type of infection identified in each municipality was determined by the detection of one or more of the agents investigated by IHC and/or molecular testing. Accordingly, infections within the municipalities were classified as being associated with MCFV, OvGHV2, as well as the other agents investigated. Cattle from municipalities were classified as being infected by OvGHV2 due to the amplification of nucleic acids with or without the simultaneous detection of MCFV tissue antigens within the lungs. Alternatively, infection was considered MCFV-related due to the detection of tissue antigens without the simultaneous amplification of OvGHV2 DNA in these pulmonary samples.

Additionally, infections were considered singular when only one infectious disease agent was detected by a particular investigation at each municipality. Similarly, infections were mixed/simultaneous when more than one of these agents was identified concomitantly at the municipalities.

### 2.5. Spatial Mapping of Municipalities Within Mato Grosso

Data on the municipal boundaries of the state of Mato Grosso were obtained from the IBGE [36] and processed in QGIS 3.28. Information on cases of cattle with IP by municipality was organized in CSV format and associated with the vector layer by means of a spatial join.

For the spatial representation, graduated symbology was applied with classification in Quantiles, distributing the values in classes with colors ranging from light to dark, according to the number of cases. The map was prepared in the SIRGAS 2000/UTM zone 21S reference system (EPSG: 31981) in the QGIS Layout.

### 2.6. Statistical Analyses

Statistical analyses were performed using the open-access R statistical software (v4.4.1 R Core Team 2024). The Fisher’s exact test, Pearson’s Chi-squared test, and the Wilcoxon rank sum test were used to determine (a) possible risk factors for the occurrence of OvGHV2 and MCFV; and (b) the possible associations between the occurrence of pneumonia and several variables (sex, infectious agents, mesoregion, production system, and seasonality). The *p*-value ≤ 0.05 was considered significant. Additionally, when necessary, descriptive statistics were used to present other relationships observed during this investigation.

## 3. Results

### 3.1. Histopathological and Immunohistochemical Findings

**Figure 1:**
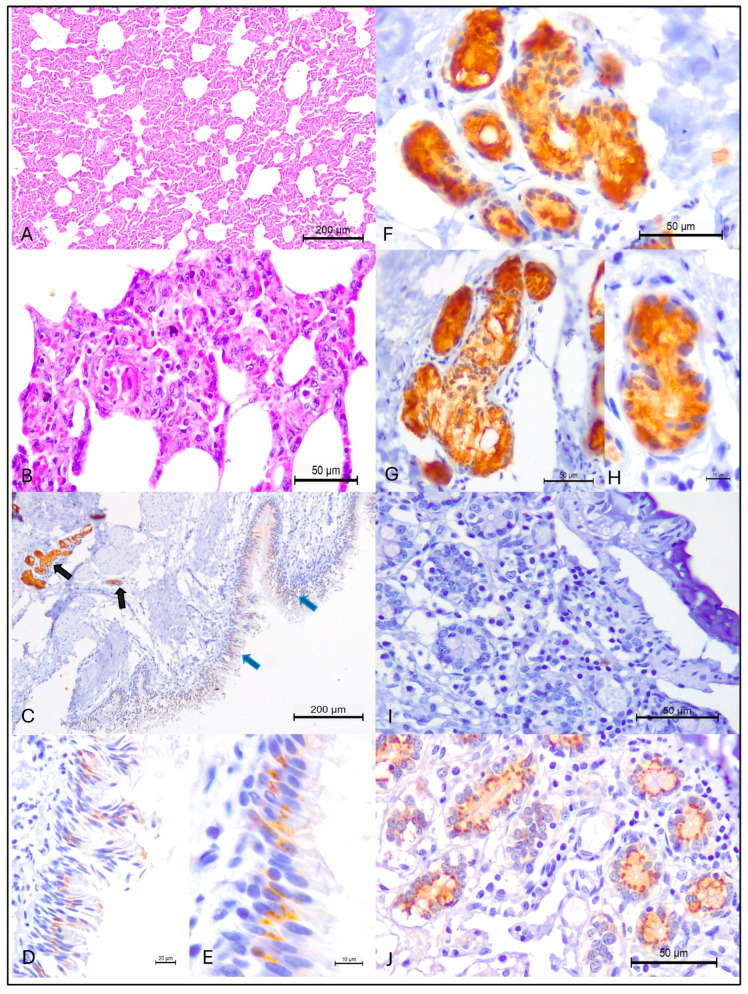
Histopathological and immunohistochemical findings observed in beef cattle from Mato Grosso, Brazil. Interstitial pneumonia is shown at (**A**,**B**). Observe positive intracytoplasmic immunoreactivity to MCFV antigens within bronchial (blue arrows) and peribronchial glandular (black arrows) of the lungs (**C**); with closer views of the intracytoplasmic accumulation of MCFV antigens within bronchial (**D**,**E**) and peribronchial glandular (**F**–**H**). The negative (**I**) and positive (**J**) controls are provided. (**A**,**B**), Hematoxylin and eosin stain; (**C**–**J**), Immunoperoxidase counterstained with Hematoxylin. Bars, (**A**,**C**), 200 µm; (**B**,**F**,**G**,**I**,**J**), 50 µm; (**D**), 20 µm; (**H**), 10 µm.

**Figure 2:**
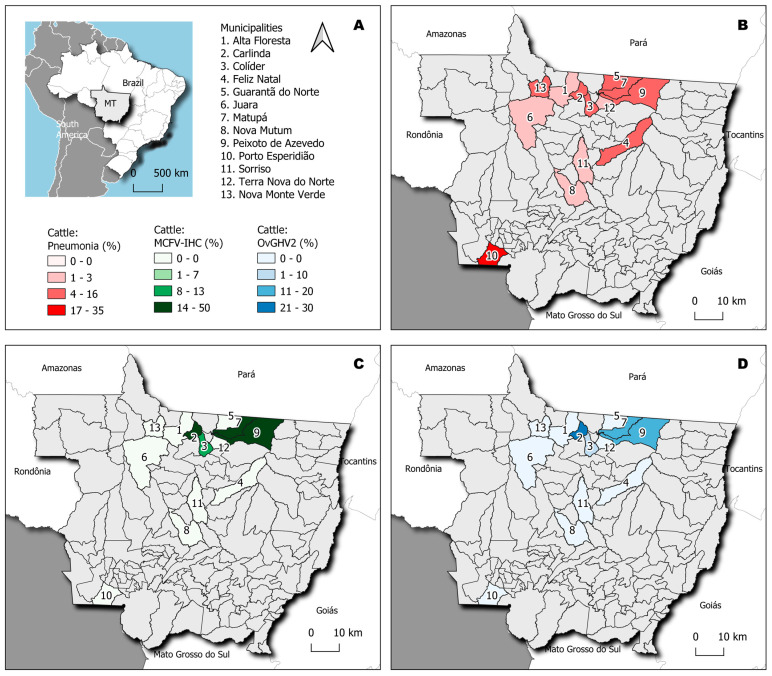
Geographical representation of the municipalities within the State of Mato Grosso, Brazil. The state of Mato Grosso (MT) is highlighted (**A**), and the frequency of cattle with interstitial pneumonia (IP) in each municipality is shown (**B**). Compare the municipalities in which the detection of MCFV antigens in cattle with IP occurred (**C**) with those where OvGHV2 was amplified (**D**).

Histopathological evaluation revealed that pneumonia occurred in 98% (43/44) of the lungs evaluated, with IP being the only histological pattern identified in these animals. IP was confirmed by the thickening of pulmonary alveolar septa due to the proliferation of type II pneumocytes with influx of lymphoplasmacytic inflammatory cells without neutrophilic exudate within the lumens of alveoli, bronchus, and bronchioles (Figure 1A,B). Furthermore, IP was diagnosed in cattle from all municipalities evaluated (Table 1), being more frequently identified in animals from the municipalities of Porto Esperidião (35%; 15/43) and Peixoto de Azevedo (14%; 6/43). The geographical locations of the municipalities containing cattle with IP are shown in Figure 2B.

The IHC analysis revealed positive intracytoplasmic immunoactivity to MCFV antigens in 37% (16/34) of the lungs with IP. Intralesional antigens of MCFV were detected within epithelial cells of the bronchus and peribronchial glands (Figure 1C–H). Additionally, MCFV antigens were more frequently detected in cattle from Porto Esperidião (50%; 8/16) relative to animals from the other municipalities evaluated (Table 1; Figure 2C). Within this specific municipality, MCFV antigens were detected in more than 50% (8/15) of all cattle with IP. Furthermore, MCFV antigens were detected in two of the three animals from the municipality of Guarantã do Norte, being the only pathogen identified in cattle from this municipality with IP (Figure 2).

### 3.2. Molecular Detection of Respiratory Pathogens in Cattle with Interstitial Pneumonia

Only the nucleic acids of OvGHV2, BCoV, BVDV, and *M. haemolytica* were amplified from their respective molecular assays. Direct sequencing confirmed the amplicons detected; the OvGHV2 strain detected during this investigation is named OvGHV2/BRA-UEL/MT-738/2024 and is deposited in GenBank (Accession # PX056706).

The absolute occurrences of the four agents in cattle with IP are provided in Table 1; infections were more frequently due to OvGHV2 (23%; 10/43), followed by BVDV (5/43), BCoV (12%; 3/43), and *M. haemolytica* (2%; 1/43). Furthermore, the nucleic acids of BRSV, BoAHV1, BPIV3, *P. multocida*, *H. somni*, and *M. bovis* were not amplified during their respective assays.

Infections due to OvGHV2 occurred in cattle from 38% (5/13) of the municipalities that had animals with IP (Table 1), being more frequently detected in cattle from Carlinda (30%; 3/10), followed by 20% (2/10) in those from Matupá, Peixoto de Azevedo, and Porto Esperidião (Figure 2D). Most (60%; 3/5) of the pulmonary infections associated with BVDV occurred in cattle from Porto Esperidião. The three pulmonary infections due to BCoV were equally distributed in cattle from the municipalities of Nova Monte Verde, Peixoto de Azevedo, and Porto Esperidião. The sole pulmonary infection attributed to *M. haemolytica* was identified in an animal from Colíder.

### 3.3. Occurrence of Singular and Concomitant Pulmonary Infections in Cattle from the Municipalities of Mato Grosso

During this investigation, most (69%; 9/13) of the municipalities with cattle diagnosed with IP (Table 1) contained at least one animal that was infected by one of the five pathogens (MCFV, OvGHV2, BCoV, BVDV, and *M. haemolytica*). Additionally, animals from the municipalities of Peixoto de Azevedo and Porto Esperidião with IP were more severely affected due to the occurrence of quadruple infections involving MCFV, OvGHV2, BCoV, and BVDV. Singular pulmonary infections were only due to OvGHV2 (*n* = 2) and MCFV (*n* = 3). These infections occurred in cattle from the municipalities of Carlinda and Matupá (OvGHV2), Guarantã do Norte, Sorriso, and Terra Nova do Norte (MCFV). Dual infections were associated with OvGHV2, being diagnosed in cattle from the municipalities of Colíder (OvGHV2 and *M. haemolytica*) and Nova Monte Verde (OvGHV2 and BCoV). Additionally, the nucleic acids of none of the 10 pathogens of BRD evaluated were amplified from the lungs of cattle with histological evidence of IP from the municipalities of Alta Floresta, Feliz Natal, Juara, and Nova Mutum. All cattle from these municipalities were reared under the semi-intensive production system.

### 3.4. Distribution of Interstitial Pneumonia and Pathogens Within the Production Rearing Systems

The frequency of occurrence of IP and the detection of pathogens by IHC and molecular biology is graphically represented in Figure 2. Comparatively, the frequency of IP, as well as the identification of all pathogens, was comparatively more elevated in cattle reared under the semi-intensive system as compared to those maintained within the intensive and extensive rearing of cattle within the municipalities of MT.

**Figure 3:**
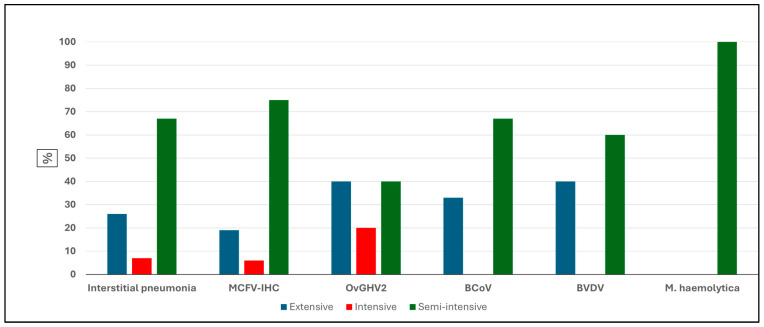
Comparative frequencies of interstitial pneumonia and pulmonary pathogens detected in beef cattle based on the production system. Legend: MCFV-IHC, malignant catarrhal fever virus immunohistochemistry; OvGHV2, ovine gammaherpesvirus 2; BCoV, bovine coronavirus; BVDV, bovine viral diarrhea virus.

Curiously, BCoV, BVDV, and *M. haemolytica* were not detected in the lungs of cattle with IP that were reared under the intensive production system (Figure 3). Additionally, *M. haemolytica* was only identified in the lungs of cattle with IP reared under the semi-intensive system, as compared to being undetected in cattle with IP but reared under the extensive and intensive beef cattle production systems.

### 3.5. Risk Factors Associated with Infections by MCFV and OvGHV2 in Beef Cattle from Mato Grosso

**Figure 4:**
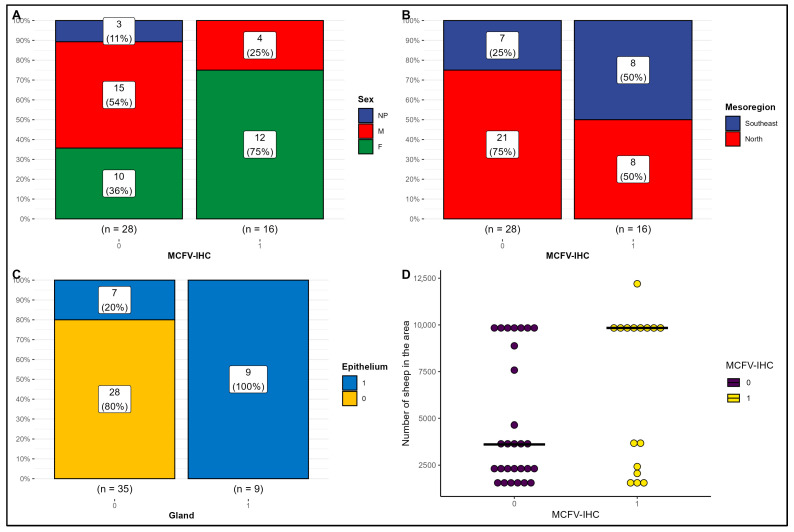
Risk factors for the detection of malignant catarrhal fever virus (MCFV) antigens in beef cattle with interstitial pneumonia from Mato Grosso, Brazil. Observe the significant association (*p* = 0.048) between the sex of the infected cattle and the detection of MCFV antigens (**A**) and the positive correlation (*p* ≤ 0.001) between the occurrence of MCFV antigens within bronchial epithelial cells and peribronchial glands (**C**). There was no association between the detection of MCFV antigens relative to the mesoregion of origin (*p* = 0.092; (**B**)) or the total number of sheep (*p* = 0.13; (**D**)).

The statistical analyses using the combined Fisher’s exact, Pearson’s Chi-squared, or Wilcoxon rank sum tests to determine the possible association between the detection of MCFV antigens in cattle with IP are provided in Table 2 and graphically represented in Figure 3. These analyses revealed that there was a significant difference (*p* = 0.048) between the detection of MCFV antigens in the lungs of female cows (75%; 12/16) with IP relative to male animals (25%; 4/16) during this investigation (Figure 4A). Alternatively, there was no association (*p* = 0.111) between the detection of MCFV antigens and the mesoregions of origins of these animals (Figure 4B). However, there was a positive association (*p* < 0.001) between the IHC detection of MCFV antigens within the bronchial epithelium and the epithelium of peribronchial glands of cattle with IP (Figure 4C). When there is a positive immunoreactivity within the peribronchial glands of cattle with IP, the bronchial epithelium will likely be always positive, whereas when there is no positive immunoreactivity within the glandular epithelium, the risk of positive immunoreactivity within the bronchial epithelium is only 25%.

Although there was no statistical difference (*p* = 0.3) between the occurrence of MCFV antigens relative to the sheep: cattle ratio (SCR) in cattle with IP (Table 2; Figure 4D), the population of cattle with detectable antigens of MCFV, were reared in areas that contained 9837 sheep, as compared with cattle without detectable MCFV antigens reared in areas having 3611 sheep (*p* = 0.13).

In contrast, the detection of MCFV antigens was not significantly associated with the development of IP (*p* = 0.9), infection by OvGHV2 (>0.9), the mesoregion of origin of the affected cattle (*p* = 0.09), the production system (*p* = 0.8), and the season (*p* = 0.07) during which detection occurred.

### 3.6. The Epidemiology of Pneumonia in Beef Cattle from Some Municipalities of Mato Grosso

The epidemiological data of the occurrence of IP in cattle during this investigation are provided in Supplementary Table S2; no statistical association was detected with the variables analysed. Nevertheless, IP was more frequently identified in female (49%; 21/43) relative to male (44%; 19/23) cattle, while the sex of the animals was not reported in 7% (3/43) of these. When the pathogens of BRD were compared (as described above), OvGHV2 was more frequently detected in cattle with IP, occurring in 23% (10/23) of all cases, followed by BVDV (12%; 5/43), BCoV (7%; 3/43), and *M. haemolytica* (2%; 1/43). MCFV antigens were detected in 63% (27/43) of the lungs of cattle with IP. Additionally, IP was more frequently diagnosed in cattle from the Northern mesoregion (65%; 28/43) of MT as compared to those from the Southeastern mesoregion (35%; 15/43). Moreover, IP was diagnosed in cattle reared within all production systems, with those reared semi-intensively (67%; 29/43) being more frequently diagnosed with this pattern of pulmonary alteration. Furthermore, seasonality had no direct relationship (*p* = 0.5) on the occurrence of IP, with comparatively more cattle being diagnosed during spring (53%; 23/43), relative to winter (42%; 18/43) and autumn (5%; 2/43).

## 4. Discussion

This is the first study to provide information as to the occurrence of pulmonary disease in beef cattle from several municipalities of MT; a previous investigation described the serological profile of steers on entering feedlots from MT relative to the occurrence of BVDV, BPIV3, BRSV, and BoAHV1 [29]. Consequently, the results of this investigation are of fundamental importance as it provides an overview of the occurrence of pulmonary disease in beef cattle reared in the principal cattle producing region of Brazil, and adds to the few studies that have previously diagnosed OvGHV2-induced diseases in cattle [25,26,27] and a brocket deer [37] from this state and within the Cerrado biome of Brazil. Beef cattle production in Brazil occurs primarily within four of the six biomes, with the Cerrado biome having the largest population of cattle [23]. Although these results are based on a limited number of samples from only 13 municipalities of MT, and hence not representative of the entire state, these initial findings provide a spotlight on the occurrence of pulmonary disease of cattle within this state and the Cerrado biome.

In this study, the detection of pulmonary injury was confirmed by the histopathological analysis of lung fragments, where almost all cattle evaluated demonstrated IP. Additionally, the detection of MCFV antigens as well as the nucleic acids of OvGHV2, BVDV, BCoV, and *M. haemolytica* in the lungs of these animals with IP confirmed the development of lung disease due to the detection of infectious agents within damaged pulmonary tissues [38]. Furthermore, direct sequencing confirmed the results of the amplicons in their respective molecular assays. In addition, the non-detection of the nucleic acids of BRSV, BoAHV1, BPIV3, *P. multocida*, *H. somni*, and *M. bovis* suggests that these agents of BRD were not associated with the development of the pulmonary alterations herein described. However, caution must be taken with the interpretation of the non-detection of these agents, since this may be directly related to sampling at the end of the beef rearing system.

### 4.1. OvGHV2 Was the Most Frequently Identified Pathogen Associated with Pulmonary Disease

During this investigation, OvGHV2 was the most frequently identified pathogen associated with the development of IP, occurring in singular and concomitant infections, in cattle originating from the municipalities of Carlinda, Matupá, Peixoto de Azevedo, and Porto Esperidião. In the world context, these four municipalities had 1,493,774 heads of cattle in 2023 [24], and a land mass equivalent to that of Belgium, Armenia, or Albania.

Previous studies have shown that OvGHV2 was associated with the development of pulmonary impairment during an outbreak of acute respiratory disease in dairy cattle [18], in calves with histological evidence of pulmonary disease [33,39], as well as in sucking calves [17]. It must be highlighted that in most of these cases [18,33,39], OvGHV2 antigens were detected by IHC within pulmonary tissues, confirming the development of disease [38]. Furthermore, infections due to OvGHV2 may be a possible risk factor for the occurrence of respiratory infections induced by *Mycoplasmopsis bovirhinis* in suckling dairy calves [16]. Alternatively, no risk factor was associated with the development of OvGHV2-induced pulmonary disease during this study. Additionally, a MCFV, most likely OvGHV2, was associated with the development of BRD in dairy and beef cattle [40]. Collectively, there is adequate documented evidence to support the theory that OvGHV2 should be considered as a possible cause of pulmonary disease in cattle [7], and hence, must be included in the differential diagnosis of cattle with BRD.

In three animals from distinct municipalities, there was the detection of MCFV antigens within the lungs of cattle with histological evidence of IP but without the concomitant detection of OvGHV2 DNA. These conclusions resulted in the diagnosis of MCFV-associated lung disease; similar findings were described in adult cattle [11], a dairy calf [41], one bovine fetus [10], and in subclinically infected wild boars [42] with histological demonstration of pulmonary disease. Furthermore, during this investigation, female cattle with IP were at a significantly higher risk of having MCFV detectable antigens relative to male animals. Moreover, the results of this study revealed that the detection of MCFV antigens can occur independently either within epithelial cells of the bronchus or the peribronchial glands in cattle with IP, indicating that the occurrence of MCFV antigens within these two histologic locations is adequate to establish a diagnosis of this infection. These findings are in accordance with previous studies that have identified MCFV antigens frequently within these histological locations of the lungs of cattle with BRD [11,40,41]. Therefore, this is additional evidence to associate the contribution of *Macavirus* with the occurrence of pulmonary diseases in mammals.

Curiously, in five animals from four different municipalities that were reared on the semi-intensive production system, none of the infectious agents investigated were observed, suggesting that these pathogens were not associated with the development of IP in these animals. Accordingly, the participation of other agents of IP that were not evaluated during this study, as well as the occurrence of AIP [20] cannot be completely ignored. Moreover, AIP is more frequently identified in female relative to male animals [20,43], as was observed during this investigation. However, clinical data is necessary to confirm AIP in the current cases. Additionally, as indicated above, the sampling of beef cattle at the end of the production cycle could have contributed towards the results herein described.

### 4.2. OvGHV2-Induced Infections and Pulmonary Disease May Be Frequent in Cattle from These Municipalities of Mato Grosso

Since the diagnosis of IP with the subsequent detection of OvGHV2 in cattle that originated from four distinct municipalities only occurred after slaughter, it can be inferred that these animals were subclinically infected [38]. This is because all cattle were approved for slaughter during routine inspection at each slaughterhouse, where clinically impaired animals would have been removed. Furthermore, OvGHV2-associated subclinical infections in cattle from Brazil seem to be more frequent than in other geographical locations [30], suggesting that the epidemiology of this pathogen in this continental nation may be somewhat different. Therefore, the occurrence of subclinical OvGHV2-associated infections in beef cattle may be more widespread in this state than was previously reported. Nevertheless, an investigation involving a larger population of animals from more municipalities of MT will be required to understand the dynamics of pulmonary disease within this state.

During this study, it was not known exactly if all cattle were reared at establishments that contained sheep on the premises. However, the sheep: cattle ratio (SCR) had no effect on the development of IP, the detection of MCFV antigens, or infections by OvGHV2. The SCR was established as an indicator for the possible occurrence of clinical SA-MCF in geographical regions where cattle are reared concomitantly with sheep [44]. The extremely reduced SCR of the state of MT is directly related to the comparatively reduced number of sheep reared in this state, considering that in 2023, MT contributed to only 2% (394,737/21,792,139) of all sheep reared in Brazil, while being the home to the largest population of cattle nationwide [24]. Although there was no positive association between the SCR and the occurrence of infections due to OvGHV2 and MCFV during this investigation, there is numerical and visual evidence to suggest that the SCR and sheep number may be associated with the occurrence of these infections. However, the reduced number of samples during this investigation was probably not adequate to establish a statistical association. Furthermore, in all previously reported outbreaks of SA-MCF in cattle from the state of MT [25,26,27], sheep were reared concomitantly with the affected animals, and with the brocket deer [37].

As indicated above, the findings from this investigation represent the only study that provides information as to the occurrence of pneumonia in cattle from this state, with IP being predominantly identified in subclinically infected cattle from the 13 municipalities. A serological evaluation done with steers on entering feedlots in MT revealed seropositivity of 43% (95/222) for BVDV, as well as elevated seropositivity for BoAHV1, BRSV, and BRSV [29], suggesting that cattle from this state had previous contact with agents known to induce IP. Curiously, in a retrospective nine-year study based on the number of samples received and post-mortem evaluations of cattle from a veterinary diagnostic laboratory, pneumonia was diagnosed in only 2% (23/1124) of all cattle evaluated in MT [28]. Alternatively, pulmonary impairment [25] with elevated viral loads of OvGHV2 [45] were described in an outbreak of SA-MCF in cattle from this state. While additional data relative to the occurrence of pulmonary disease in cattle from MT was not detected in major English (e.g., PubMed, Scopus, Web of Science) and Latin (e.g., SciELO) databases. Therefore, more information as to the occurrence of pulmonary disease is required to fully understand the dynamics within this high-producing cattle region of Brazil.

Finally, the occurrence of concomitant infections in cattle with pulmonary impairment from several municipalities of MT is consistent with the multietiological nature of BRD in Brazil [12] and elsewhere [15,19]. However, during this investigation, risk factors associated with the development of pneumonia in cattle from the 13 municipalities were not determined. Additionally, the participation of OvGHV2 in all simultaneous infections during this investigation is another indicator of the probable role of this pathogen in the development of pulmonary disease in cattle. These findings are in accord with another study during which OvGHV2 was one of the most frequently identified respiratory pathogens in dairy calves, and was associated with dual, triple, and quadruple infections [17].

### 4.3. Study Limitations

This study had two major setbacks, which could have provided a better understanding of the data collected. Firstly, the clinical status of the herd of origin of all animals remains unknown. Knowledge of the clinical status at these farms would have provided information as to the occurrence of clinical manifestations of respiratory disease, since the animal slaughtered may not be a true representation of the herd status. Secondly, the evaluation of larger populations of cattle would have provided a better overview of the occurrence of pulmonary disease within the state of MT. Notwithstanding the above, the data obtained in this study is a landmark for the diagnosis and the understanding of pulmonary diseases in cattle from this state.

## 5. Conclusions

Interstitial pneumonia (IP) was the predominant pattern (98%; 43/44) of pulmonary lesion observed in lung sections of 44 beef cattle that originated from 13 municipalities within the state of Mato Grosso. Intralesional MCFV antigens were detected in 37% (16/43) of the lungs with IP, while OvGHV2 DNA was amplified from 23% (10/43) of these. Additionally, the nucleic acids of BVDV, BCoV, and *M. haemolytica* were also detected in the lungs with IP. Singular infections due to MCFV and OvGHV2 were identified, while OvGHV2 participated in all simultaneous infections. These findings suggest that pneumonia may be more frequent in cattle from the state of MT than was previously reported, provide an overview of the occurrence of pulmonary disease in beef cattle from the major cattle-producing state of Brazil, and offer additional evidence of the possible role of OvGHV2 in the development of pulmonary disease in cattle.

## Figures and Tables

**Table 1:** Distribution of the absolute occurrences of interstitial pneumonia, with the immunohistochemical detection of malignant catarrhal fever virus (MCFV) antigens, molecular amplification of infectious agents, and the type of infections identified in beef cattle from municipalities of Mato Grosso, Brazil.

Municipalities	Cattle with Interstitial Pneumonia	MCFV IHC	Pathogens Detected by Molecular Assays ^1^				Type of Infections
			OvGHV2	BCoV	BVDV	MH	
Alta Floresta	1	0	0	0	0	0	None
Carlinda	4	1	3	0	0	0	Singular; OvGHV2
Colíder	3	0	1	0	0	1	Mixed; OvGHV2 + MH
Feliz Natal	2	0	0	0	0	0	None
Guarantã do Norte	3	2	0	0	0	0	Singular; MCFV
Juara	1	0	0	0	0	0	None
Matupá	3	1	2	0	0	0	Mixed; OvGHV2 + MCFV
Nova Monte Verde	2	1	0	1	0	0	Mixed; MCFC + BCoV
Nova Mutum	1	0	0	0	0	0	None
Peixoto de Azevedo	6	1	2	1	2	0	Quadruple; OvGHV2 + BCoV + BVDV
Porto Esperidião	15	8	2	1	3	0	Quadruple; OvGHV2 + BCoV + BVDV
Sorriso	1	1	0	0	0	0	Singular; MCFV
Terra Nova do Norte	1	1	0	0	0	0	Singular; MCFV
Total	43	16	10	3	5	1	

Legend: MCFV-IHC, malignant catarrhal fever virus immunohistochemistry; OvGHV2, ovine gammaherpesvirus 2; BCoV, bovine coronavirus; BVDV, bovine viral diarrhea virus; MH, *Mannheimia haemolytica*; ^1^, only results of positive molecular assays are shown.

**Table 2:** Risk factors associated with the detection of malignant catarrhal fever virus antigens in beef cattle from Mato Grosso, Brazil.

Variables	Negative ^1^	Positive ^1^	*p*-Value ^2^
	N = 281	N = 161	
Pneumonia	27 (96%)	16 (100%)	>0.9
Sex			0.048
Female	10 (36%)	12 (75%)	
Male	15 (54%)	4 (25%)	
Not Provided	3 (11%)	0 (0%)	
Ovine gammaherpesvirus 2	6 (21%)	4 (25%)	>0.9
Mesoregion			0.092
North	21 (75%)	8 (50%)	
Southeast	7 (25%)	8 (50%)	
Production system			0.8
Extensive	9 (32%)	3 (19%)	
Intensive	2 (7%)	1 (6%)	
Semi-intensive	17 (61%)	12 (75%)	
Season			0.073
Autum	1 (3.6%)	1 (6.3%)	
Spring	18 (64%)	5 (31%)	
Winter	9 (32%)	10 (63%)	
Cattle	386,284 (234,667, 663,778)	530,494 (324,685, 663,778)	0.6
Sheep	3611 (2205, 9358)	9837 (2242, 9837)	0.13
SCR	0.009 (0.006, 0.015)	0.015 (0.007, 0.015)	0.3

^1^ n (%); Median (Q1, Q3); ^2^ Fisher’s exact test; Pearson’s Chi-squared test; Wilcoxon rank sum test.

## Data Availability

The nucleotide sequence of the OvGHV2 identified during this study is deposited in GenBank (https://www.ncbi.nlm.nih.gov/genbank// accessed on 31 July 2025).

## Supplementary Materials

The following supporting information can be downloaded at: https://www.mdpi.com/article/10.3390/pathogens14090945/s1: Supplementary Table S1. List of primers; Supplementary Table S2. Epidemiology of pneumonia in Mato Grosso, Brazil.
