# Supplementary material for: The Association of Macavirus and Ovine Gammaherpesvirus 2 with Pneumonia in Beef Cattle from Mato Grosso, Brazil
Source: Pathogens. 2025 Sep 18;14(9):945. doi: 10.3390/pathogens14090945 (PMC12473052; doi:10.3390/pathogens14090945)
Supplement: Supplementary file 1 [file pathogens-14-00945-s001.zip › Supplementary Table S1 List of primers 04-08-25.pdf]

Supplementary Table S1. Targets genes, primers, and amplicon size of the molecular assays used to identify infectious disease pathogens of diseases of cattle.

| Pathogens | Target genes | Primer sequences (5′ – 3′) | Amplicons size (bp) | Reference |
|---|---|---|---|---|
| | | **Viral** | | |
| OvGHV2 | Tegument protein | Fw – AGTCTGGGTATATGAATCCAGATGGCTCTC<br>Rv - AAGATAAGCACCAGTTATGCATCTGATAAA<br>Rv – TTCTGGGGTAGTGGCGAGCGAAGGCTTC | 422<br>238 | 1 |
| BCoV | N gene | Fw - CGATGAGGCTATTCCGAC-<br>Rv – TGTGGGTGCGAGTTCTGC<br>Fw - TTGCTAGTCTTGTTCTGGC | 454<br>251 | 2 |
| BVDV | 5′UTR | Fw – ATGCCCWTAGTAGGACTAGCA<br>Rv – TCAACTCCATGTGCCATGTAC | 288 | 3 |
| BRSV | Glycoprotein G | Fw - CCACCCTAGCAATGATAACCTTGAC<br>Rv - AAGAGAGGATGCYTTGCTGTGG<br>Fw - CATCAATCCAAAGCACCACACTGTC<br>Rv - GCTAGTTCTGTGGTGGATTGTTGTC | 603<br>371 | 4 |
| BoAHV1 | Glycoprotein C | Fw - CAACCGAGACGGAAAGCTCC<br>Rv- AGTGCACGTACAGCGGCTCG | 354 | 5 |
| BPI3 | HN gene | Fw - GAATGACTCATGATAGAGGTAT<br>Rv - AGGACAACCAGTTGTATTACAT | 647 | 6 |
| | | **Bacterial** | | |
| *Mannheimia haemolytica* | lktA-artJ intergenic region | Fw - GTCCCTGTGTTTTCATTATAAG<br>Rv - CACTCGATAATTATTCTAAATTAG | 385 | 7 |
| *Histophilus somni* | 16S rDNA | Fw - GAAGGCGATTAGTTTAAGAG<br>Rv - TTCGGGCACCAAGTRTTCA | 400 | 8 |
| *Pasteurella multocida* | ORF KMT1 clone | Fw – GCTGTAAACGAACTCGCCAC<br>Rv - ATCCGCTATTTACCCAGTGG | 460 | 9 |
| *Mycoplasma bovis* | ITS region | Fw - CCGTCAAACYATGGGAGC<br>Rv – GTGYCCCGCCMTACTCAGG<br>Fw - GTACACTTGTCTTTTATCACTATA<br>Rv - AAGGTATCTCGCTTTATGTCCT | 864<br>488 | 10 |

## References

1. Baxter SI, Pow I, Bridgen A, Reid HW (1993) PCR detection of the sheep- associated agent of malignant catarrhal fever. Arch Virol 132: 145- 159. doi:10.1007/bf01309849.

2. Takiuchi E, Stipp DT, Alfieri AF, Alfieri AA (2006) Improved detection of bovine coronavirus N gene in faeces of calves infected naturally by a seminested PCR assay and an internal control. J Virol Methods 131: 148- 154. doi: 10.1016/j.jviromet.2005.08.005.

3. Vilcek S, Herring AJ, Herring JA, Nettleton PF, Lowings JP, Paton DJ (1994) Pestiviruses isolated from pigs, cattle and sheep can be allocated into at least three genogroups using polymerase chain reaction and restriction endonuclease analysis. Arch Virol 136: 309- 323. doi: 10.1007/bf01321060.

4. Vilcek S, Elvander M, Ballagi- Pordany A, Belak S (1994) Development of nested PCR assays for detection of bovine respiratory syncytial virus in clinical samples. J Clin Microbiol 32: 2225-2231. doi: 10.1128/jcm.32.9.2225- 2231.1994.

5. Claus MP, Alfieri AF, Folgueras- Flatschart AV, Wosiacki SR, Médici KC, Alfieri AA (2005) Rapid detection and differentiation of bovine herpesvirus 1 and 5 glycoprotein C gene in clinical specimens by multiplex- PCR. J Virol Methods 128: 183- 188. doi: 10.1016/j.jviromet.2005.05.001.

6. Zhu YM, Shi HF, Gao YR, Xin JQ, Liu NH, Xiang WH, Ren XG, Feng JK, Zhao LP, Xue F (2011) Isolation and genetic characterization of bovine parainfluenza virus type 3 from cattle in China. Vet Microbiol 149: 446- 451. doi: 10.1016/j.vetmic.2010.11.011.

7. Angen O, Thomsen J, Larsen LE, Larsen J, Kokotovic B, Heegaard PM, Enemark JM (2009) Respiratory disease in calves: microbiological investigations on trans- tracheally aspirated bronchoalveolar fluid and acute phase protein response. Vet Microbiol 137: 165- 171. doi: 10.1016/j.vetmic.2008.12.024.

8. Angen O, Ahrens P, Tegtmeier C (1998) Development of a PCR test for identification of Haemophilus somnus in pure and mixed cultures. Vet Microbiol 63: 39- 48. doi: 10.1016/s0378- 1135(98)00222- 3.

9. Townsend KM, Frost AJ, Lee CW, Papadimitriou JM, Dawkins HJ (1998) Development of PCR assays for species- and type- specific identification of Pasteurella multocida isolates. J Clin Microbiol 36: 1096-1100. doi: 10.1128/jcm.36.4.1096-1100.1998.

10. Voltarelli DC, de Alcântara BK, Lunardi M, Alfieri AF, de Arruda Leme R, Alfieri AA. (2018) A nested-PCR strategy for molecular diagnosis of mollicutes in uncultured biological samples from cows with vulvovaginitis. Anim Reprod Sci 188:137- 143, doi: 10.1016/j.anireprosci.2017.11.018.
