# Supplementary material for: The Association of Macavirus and Ovine Gammaherpesvirus 2 with Pneumonia in Beef Cattle from Mato Grosso, Brazil
Source: Pathogens. 2025 Sep 18;14(9):945. doi: 10.3390/pathogens14090945 (PMC12473052; doi:10.3390/pathogens14090945)
Supplement: Supplementary file 1 [file pathogens-14-00945-s001.zip › Supplementary Table S2 Epidemiology of IP in Mato Grosso 04-08-25.pdf]

pathogens

Supplementary Table S2. Epidemiological features of the occurrence of interstitial pneumonia in beef cattle from Mato Grosso, Brazil.

Variables	0<br>N=1[1]	1<br>N=43[1]	*p*-value
Sex			>0.9
Female	1 (100%)	21 (49%)	
Male	0 (0%)	19 (44%)	
Not provided	0 (0%)	3 (7%)	
Infectious agents			
Ovine gammaherpesvirus 2	0 (0%)	10 (23%)	>0.9
Bovine alphaherpesvirus 1	1 (100%)	43 (100%)	
Bovine viral diarrhea virus	0 (0%)	5 (12%)	>0.9
Bovine parainfluenza virus 3	1 (100%)	43 (100%)	
Bovine respiratory syncytial virus	1 (100%)	43 (100%)	
Bovine coronavirus	0 (0%)	3 (7%)	>0.9
Pasteurella multocida	1 (100%)	43 (100%)	
Histophilus somni	1 (100%)	43 (100%)	
Mannheimia haemolytica	0 (0%)	1 (2%)	>0.9
Mycoplasmopsis bovis	1 (100%)	43 (100%)	
Malignant catarrhal fever virus-immunohistochemistry			>0.9
	1 (100%)	27 (63%)	
	0 (0%)	16 (37%)	
Mesoregion			>0.9
North	1 (100%)	28 (65%)	
Southeast	0 (0%)	15 (35%)	
Production system			0.3
Extensive	1 (100%)	11 (26%)	
Intensive	0 (0%)	3 (7%)	
Semi-intensive	0 (0%)	29 (67%)	
Season			0.5
Autum	0 (0%)	2 (5%)	
Spring	0 (0%)	23 (53%)	
Winter	1 (100%)	18 (42%)	
Cattle	347,789 (347,789, 347,789)	397,209 (234,667, 663,778)	0.5
Sheep	3,678 (3,678, 3,678)	3,678 (2,205, 9,837)	>0.9
Sheep: Cattle Ratio	0.011 (0.011, 0.011)	0.011 (0.006, 0.015)	>0.9

[1] n (%); Median (Q1, Q3)

[2] Fisher's exact test; Wilcoxon rank sum test
